# Supplementary material for: Screening Digitaria eriantha cv. Suvernola Endophytic Bacteria for Maize Growth Promotion
Source: Plants (Basel). 2023 Jul 8;12(14):2589. doi: 10.3390/plants12142589 (PMC10385894; doi:10.3390/plants12142589)
Supplement: Supplementary file 1 [file plants-12-02589-s001.zip › Supplementary table S1.pdf]

Supplementary table S1. *In vitro* growth promotion evaluation. BNF – growth in N free media. IAA – IAA synthesis. SI – calcium phosphate solubilization index. Siderophore – siderophore syntehesis.

Strain	Genus	BNF	IAA	SI	Siderophore
			μg mL^{-1}		%
5038		-	95.94	-	28.60
5068	*Enterobacter* sp.	-	19.72	1.60	10.70
5095	*Enterobacter* sp.	-	95.03	3.11	62.78
5117	*Rhizobium* sp.	+	110.77	2.49	20.16
5120	*Stenotrophomonas* sp.	+	89.53	-	39.08
5132	*Stenotrophomonas* sp.	-	25.77	-	-
5155	*Rhizobium* sp.	+	206.51	-	17.55
5167		-	33.50	-	16.39
5170	*Burkholderia* sp.	+	32.97	-	50.35
5173	*Variovorax* sp.	+	20.18	-	5.35
5177	*Agrobacterium* sp.	+	123.91	-	20.38
5183	*Rhizobium* sp.	+	118.09	-	22.06
5193	*Pseudomonas* sp.	+	22.92	-	-
5195		-	7.27	1.90	3.06
5200	*Stenotrophomonas* sp.	-	16.92	-	48.61
5211	*Stenotrophomonas* sp.	-	38.14	DNG	25.51
5223	*Stenotrophomonas* sp.	-	76.56	-	-
5225	*Stenotrophomonas* sp.	-	49.53	DNG	14.59
5226	*Stenotrophomonas* sp.	-	44.08	DNG	17.68
5227	*Pseudomonas* sp.	-	50.49	-	9.28
5276	*Rhizobium* sp.	+	205.50	-	-
5287	*Stenotrophomonas* sp.	-	139.53	-	6.37
5289		-	9.09	-	50.00
5290	*Pseudomonas* sp.	-	41.34	DNG	27.22
5297		-	103.71	-	58.06
5302	*Stenotrophomonas* sp.	-	13.68	-	-
5305	*Pseudomonas* sp.	+	69.84	-	29.71
5310	*Pantoea* sp.	+	115.69	1.71	24.36
5333		+	101.73	-	20.20
5347	*Stenotrophomonas* sp.	-	43.57	DNG	26.22
5396	*Stenotrophomonas* sp.	+	58.14	DNG	26.13
5410	*Rhizobium* sp.	+	139.47	-	24.36
121A		-	94.13	1.10	23.17
121B1	*Ochrobactrum* sp.	-	12.80	-	-

14C	*Variovorax* sp.	+	20.97	-	35.94
168A3	*Pantoea* sp.	+	117.89	-	20.95
188B		-	10.14	-	-
18C	*Rhizobium* sp.	+	121.99	-	25.69
18C2	*Rhizobium* sp.	+	122.32	-	35.94
192C	*Achromobacter* sp.	-	4.20	1.89	71.11
193A	*Enterobacter* sp.	-	18.53	-	2.22
195C1	*Pseudomonas* sp.	-	11.75	1.63	6.11
198A	*Rhizobium* sp.	+	120.69	-	20.42
199B2	*Ochrobactrum* sp.	-	102.38	1.69	28.34
20A2	*Stenotrophomonas* sp.	+	58.09	-	25.55
212B2	*Rhizobium* sp.	-	119.36	-	20.78
219B	*Rhizobium* sp.	-	94.43	-	-
225B	*Rhizobium* sp.	-	98.67	DNG	31.74
230A	*Rhizobium* sp.	+	133.45	-	18.66
231B1	*Priestia* sp.	-	24.39	-	25.15
252A	*Pantoea* sp.	-	90.77	1.71	17.22
258A2B	*Enterobacter* sp.	+	104.73	-	19.81
270B		-	110.84	-	-
270C		+	109.65	1.24	-
288A1A	*Enterobacter* sp.	+	114.61	-	48.54
288C	*Enterobacter* sp.	-	45.97	1.44	-
289A		-	8.04	1.74	31.39
294B	*Curtobacterium* sp.	-	9.09	-	25.11
297A	*Pseudomonas* sp.	-	20.42	-	14.46
303A2	*Agrobacterium* sp.	-	159.13	DNG	8.75
328A	*Rhizobium* sp.	+	118.20	-	16.49
331A2	*Stenotrophomonas* sp.	+	23.79	-	-
331C	*Rhizobium* sp.	-	9.53	-	-
333A	*Pantoea* sp.	+	123.68	1.84	17.29
333B	*Pantoea* sp.	+	103.77	2.04	26.22
334C1	*Pantoea* sp.	-	80.42	1.70	6.67
335B2	*Rhizobium* sp.	+	120.55	-	23.65
335C	*Rhizobium* sp.	+	53.08	2.32	-
336A	*Rhizobium* sp.	+	126.87	-	24.93
338A	*Rhizobium* sp.	-	14.03	-	-
33D	*Rhizobium* sp.	-	38.17	-	21.31
344B	*Rhizobium* sp.	-	19.91	1.55	13.48
346A2	*Pantoea* sp.	+	106.17	2.03	28.96
347E	*Stenotrophomonas* sp.	+	44.16	1.58	7.96
360B	*Rhizobium* sp.	-	38.14	-	1.19

363B	*Stenotrophomonas* sp.	-	25.12	-	18.04
36D	*Stenotrophomonas* sp.	-	105.63	-	-
374B	*Pantoea* sp.	-	12.80	1.80	82.78
376A	*Stenotrophomonas* sp.	-	16.57	1.93	25.00
377D	*Pantoea* sp.	-	40.28	2.02	-
387B2	*Bacillus* sp.	-	21.93	DNG	38.95
389B	*Pantoea* sp.	-	56.57	1.26	19.44
394D	*Rhizobium* sp.	+	120.38	-	25.15
396A	*Paenibacillus* sp.	-	68.37	-	15.47
396B		-	67.95	1.42	26.53
399B	*Pantoea* sp.	+	100.06	1.85	20.07
39B	*Enterobacter* sp.	+	126.31	2.02	20.29
400B	*Pantoea* sp.	-	17.06	1.50	-
402B		+	19.25	1.38	6.45
403B	*Rhizobium* sp.	-	99.79	-	6.11
410B		+	90.63	-	14.37
413D2	*Ochrobactrum* sp.	-	124.16	2.86	13.09
415A	*Pantoea* sp.	+	95.32	1.27	16.27
416A	*Pantoea* sp.	-	99.23	1.15	-
41C	*Enterobacter* sp.	+	106.39	1.54	19.23
425B	*Rhizobium* sp.	-	212.05	2.13	17.77
432D	*Stenotrophomonas* sp.	-	46.03	DNG	14.06
5038A	*Pantoea* sp.	-	27.13	-	32.27
5057A	*Shinella* sp.	-	81.47	-	1.59
5333A	*Achromobacter* sp.	+	13.57	-	21.40
5358A	*Paenibacillus* sp.	+	31.05	-	6.06
5378EB	*Pseudomonas* sp.	-	31.26	-	61.11
5379A	*Stenotrophomonas* sp.	-	93.99	1.40	22.22
G20	*Enterobacter* sp.	+	154.44	2.84	47.48
G37B1	*Pseudomonas* sp.	+	53.45	-	13.79
N12A	*Pantoea* sp.	+	111.48	2.17	23.47
N13A	*Pantoea* sp.	+	16.70	-	25.33
N14D	*Pseudomonas* sp.	+	43.94	-	33.91
N15B1	*Pseudomonas* sp.	+	81.19	1.48	18.70
N15D	*Herbaspirillum* sp.	+	28.40	-	14.01
N16B	*Rhizobium* sp.	-	128.96	-	34.04
N18A	*Pseudomonas* sp.	-	35.43	DNG	14.06
N18E		-	19.73	-	27.37
N1C	*Variovorax* sp.	+	36.17	-	22.46
N21F	*Stenotrophomonas* sp.	+	61.42	-	15.65
N22B	*Pseudomonas* sp.	+	38.37	-	10.65

N23B	*Pantoea* sp.	+	170.58	1.91	31.79
N27B	*Stenotrophomonas* sp.	-	30.52	-	22.15
N27D	*Pseudomonas* sp.	+	68.99	-	10.48
N28D	*Pseudomonas* sp.	+	117.04	-	13.93
N2C	*Variovorax* sp.	+	19.44	2.04	39.61
N31C	*Pseudomonas* sp.	+	85.55	1.59	10.61
N37C		+	66.99	2.48	19.14
N3C	*Rhizobium* sp.	-	27.35	1.69	24.89
N40E		+	103.06	3.10	37.62
N41A	*Kosakonia* sp.	+	67.58	-	15.08
N42A	*Agrobacterium* sp.	-	206.46	-	21.84
N44B2	*Stenotrophomo nas* sp.	+	101.93	-	18.30
N44E	*Achromobacter* sp.	+	116.68	-	5.04
N46D	*Serratia* sp.	+	115.06	-	31.21
N5G	*Burkholderia* sp.	-	22.27	1.49	30.37
N9B	*Burkholderia* sp.	-	21.34	2.35	65.34

Dng = did not grow
