# Supplementary material for: Screening Digitaria eriantha cv. Suvernola Endophytic Bacteria for Maize Growth Promotion
Source: Plants (Basel). 2023 Jul 8;12(14):2589. doi: 10.3390/plants12142589 (PMC10385894; doi:10.3390/plants12142589)
Supplement: Supplementary file 1 [file plants-12-02589-s001.zip › Supplementary table S2.pdf]

Supplementary table S2. Endophytic Pangolão grass bacteria effects on maize under low soluble iron availability.

Treatments	PH (cm)	CD (mm)	LA (cm^2)	SDM	RDM	SAN	SAP	RECI	REN	RE+C
				(mg)		mg plant $^{-1}$			%	
**121B1**	71	5,7	90	922	751	93	10	63	72	85
**192C**	74	5,5	94	1380	851	88	6	94	107	127
**212B2**	74	6,2	101	1404	799	91	7	95	109	129
**231B1**	69	6,5	84	1263	927	82	9	86	98	116
**252A**	85*	6,8	115	1703	1322	131	11	116	132	157
**331C**	77	6,6	115	1538	1217	121	10	105	119	142
**333B**	76	6,2	113	1463	1080	109	14	99	113	135
**335C**	74	6	126	1350	817	101	8	92	105	124
**338A**	56	5,1	127	802	308	69	7	55	62	74
**344B**	76	6,2	116	1300	864	115	8	88	101	120
**396B**	76	5,5	99	1066	567	87	13	72	83	98
**413D2**	79	6,3	137	1719	878	117	12	117	133	158
**432D**	78	5,6	110	1462	1067	129	9	99	113	135
**5038**	71	5,8	102	1198	760	103	7	81	93	110
**5057A**	70	6,2	121	1228	696	98	8	83	95	113
**5095**	78	6,5	107	1420	790	81	8	97	110	131
**5211**	78	5,9	115	1130	728	103	8	77	88	104
**5227**	78	6,4	119	1442	1512*	82	8	98	112	133
**5287**	68	5,4	109	1310	985	107	10	89	102	121
**5297**	72	5,3	94	1152	865	103	7	78	89	106
**5347**	68	6,7	120	1314	689	86	9	89	102	121
**5358A**	71	5,8	104	1080	840	86	7	73	84	100
**5410**	69	5,9	110	1336	900	81	6	91	104	123
**+C**	56	5,3	93	1221	729	70	6	83	95	113
**N9B**	72	5,7	104	1113	775	95	7	76	86	103
**NI**	70	6,2	112	1208	683	115	8	82	94	111
**CI**	69	6,4	112	1133	738	89	8	77	88	104
**VC**	1,5	6,2	2,4	2,3	3,4	5,9	11,5	3,7	3,6	3,5

NI = No inoculation; CI = Commercial inoculant; +C = high-solubility Fe source; PH = plant height; CD= colm diameter; LA= leaf area; SDM = shoot dry mass; RDM = dry mass of the root system; SAN = accumulation of N in aerial part; SAP = accumulation of P in the aerial part; RER = efficiency relative to the commercial inoculant; REN = non-inoculated control relative efficiency; RE+C = relative efficiency positive control. VC = coefficient of variation. All data were transformed by log10. Means followed by an asterisk differ significantly from the commercial inoculant control at 0.05 probability, by Dunnet's test.
